# Influenza vaccine effectiveness among outpatients in the US Influenza Vaccine Effectiveness Network by study site 2011‐2016

**DOI:** 10.1111/irv.12741

**Published:** 2020-04-16

**Authors:** Goundappa K. Balasubramani, Mary Patricia Nowalk, Theresa M. Sax, Joe Suyama, Emily Bobyock, Charles R. Rinaldo, Emily T. Martin, Arnold S. Monto, Michael L. Jackson, Manjusha J. Gaglani, Brendan Flannery, Jessie R. Chung, Richard K. Zimmerman

**Affiliations:** ^1^ Department of Epidemiology Graduate School of Public Health University of Pittsburgh Pittsburgh PA USA; ^2^ Department of Family Medicine, School of Medicine University of Pittsburgh Pittsburgh PA USA; ^3^ Department of Emergency Medicine School of Medicine University of Pittsburgh Pittsburgh PA USA; ^4^ Department of Pathology School of Medicine University of Pittsburgh Pittsburgh PA USA; ^5^ School of Public Health University of Michigan Ann Arbor MI USA; ^6^ Kaiser Permanente Washington Health Research Institute Seattle WA USA; ^7^ Baylor Scott & White Health Texas A&M University College of Medicine Temple TX USA; ^8^ Centers for Disease Control and Prevention Atlanta GA USA

**Keywords:** influenza, influenza vaccine, vaccine effectiveness

## Abstract

**Background:**

Influenza vaccination is recommended for all US residents aged ≥6 months. Vaccine effectiveness (VE) varies by age, circulating influenza strains, and the presence of high‐risk medical conditions. We examined site‐specific VE in the US Influenza VE Network, which evaluates annual influenza VE at ambulatory clinics in geographically diverse sites.

**Methods:**

Analyses were conducted on 27 180 outpatients ≥6 months old presenting with an acute respiratory infection (ARI) with cough of ≤7‐day duration during the 2011‐2016 influenza seasons. A test‐negative design was used with vaccination status defined as receipt of ≥1 dose of any influenza vaccine according to medical records, registries, and/or self‐report. Influenza infection was determined by reverse‐transcription polymerase chain reaction. VE estimates were calculated using odds ratios from multivariable logistic regression models adjusted for age, sex, race/ethnicity, time from illness onset to enrollment, high‐risk conditions, calendar time, and vaccination status‐site interaction.

**Results:**

For all sites combined, VE was statistically significant every season against all influenza and against the predominant circulating strains (VE = 19%‐50%) Few differences among four sites in the US Flu VE Network were evident in five seasons. However, in 2015‐16, overall VE in one site was 24% (95% CI = −4%‐44%), while VE in two other sites was significantly higher (61%, 95% CI = 49%‐71%; *P* = .002, and 53%, 95% CI = 33,67; *P* = .034).

**Conclusion:**

With few exceptions, site‐specific VE estimates aligned with each other and overall VE estimates. Observed VE may reflect inherent differences in community characteristics of the sites and highlights the importance of diverse settings for studying influenza vaccine effectiveness.

## 1 INTRODUCTION

Each year, infection with influenza causes an estimated 9.3 million to 45 million illnesses in the United States^1^ and an associated average cost of $11.2 billion.^2^ Annual vaccination is the most effective strategy for preventing influenza and reducing these burdens, as it has been shown to reduce the risk of influenza illness among the general population by 40%‐60% when the vaccine is well‐matched to the circulating viruses.^3^

The effectiveness of seasonal influenza vaccines is evaluated through the Centers for Disease Control and Prevention (CDC) US Influenza Vaccine Effectiveness Network, (US Flu VE Network or Network) which was established in the 2003‐2004 influenza season.^4^ The current Network conducts observational studies across five sites in the United States to evaluate medically attended, laboratory‐confirmed influenza and estimate vaccine effectiveness (VE) using a test‐negative design.^5^ Enrollees are outpatients seeking care for acute respiratory illness (ARI) who are tested for influenza using CDC's reverse‐transcription polymerase chain reaction (RT‐PCR) assay. VE is estimated by comparing the odds of vaccination among influenza‐positive and influenza‐negative outpatients who present with ARI.

Recent estimates of influenza VE have demonstrated varying VE across influenza subtypes.^6^ For example, vaccines tend to be more effective for preventing infection with influenza B and influenza A/H1N1 viruses compared to influenza A/H3N2.^3^ In a meta‐analysis of VE studies during the 2004‐2005 to 2014‐2015 influenza seasons, pooled VE estimates were as follows: 33% (95% confidence interval [CI] = 26%–39%) for A/H3N2 viruses, 61% (95% CI = 57%–65%) for A/H1N1 viruses, and 54% (95% CI = 46%–61%) for influenza B viruses.^6^

Vaccine effectiveness estimates against influenza may differ geographically due to characteristics of the study population, type of vaccine purchased by large healthcare organizations or pharmacies, vaccination coverage levels, and different circulating influenza strains. Studies from other countries indicate VE differences by strain/lineage, vaccine to circulating strain matching, age and birth cohort, and vaccination history.^7^, ^8^, ^9^ In an agent‐based cost‐effectiveness analysis using synthetic populations, DePasse et al showed different epidemiologic curves and different numbers of averted cases of influenza across five US counties that differed by demographic characteristics such as size, density, and age distributions of their populations.^10^

The US Flu VE Network consists of geographically and demographically distinct sites that contribute to annual estimations of influenza VE. It is unknown whether influenza VE differs across the sites that comprise the Network. In this study, we estimate site‐specific influenza VE among outpatients who presented with ARI during the 2011‐2016 influenza seasons at four of the five US Flu VE Network sites.

## 2 METHODS

Data were collected using a standardized protocol, as part of the CDC's US Flu VE Network study, for which detailed methods have been described.^11^, ^12^, ^13^, ^14^, ^15^, ^16^ IRB approval for the Flu VE Network protocol for all sites was granted. The screening criteria for an ARI were intentionally broad in order to maximize the pool of potential participants and included symptoms such as sore throat, fever/feverishness, congestion, wheezing, increased nasal secretions, body aches, and cough. Eligible participants were outpatients ≥6 months old seeking care for an ARI of less than 7‐day duration with a cough. Patients who had received antiviral medication in the 7 days prior to enrollment, were younger than 6 months old, had enrolled in the study during the previous 14 days, or had received influenza vaccine within 14 days of enrollment were not eligible for enrollment or data analysis. Consented participants provided data on demographics, symptoms and other measures of current health and well‐being, general health status, and self‐report of influenza vaccination that were collected during patient enrollment interviews. High‐risk conditions identified by International Classification of Diseases code Clinical Modification (versions 9 and 10 [ICD‐9/10]) assigned to a medical encounter during the year prior to enrollment were used to determine the presence of underlying health conditions associated with an increased risk of severe influenza.^16^, ^17^ ICD‐9/10 codes were derived from electronic medical records (EMR).

### 2.1 Sites

The US Flu VE Network was designed to include geographically diverse settings. The four sites included in this analysis were as follows: a large health maintenance organization located in and around Seattle, Washington, with a significant minority population of Asian enrollees; one site in a large university‐affiliated health system located in north central Texas with a significant minority population of Latino enrollees; one site that combined outpatient clinics from a university health system and an inner‐city health system located in Michigan with a significant minority population of black enrollees; and one site in a single large health system located in a county in Pennsylvania with one of the highest percentages in the USA of residents >65 years.

### 2.2 Influenza vaccine

The influenza vaccine strains used each year are shown in Table S1. For all five seasons, the A/H1N1 vaccine strain was the A/California/7/2009, while the A/H3N2 strain changed in all years but 2013‐2014 and 2014‐2015. The trivalent influenza vaccine contained a B/Victoria virus (B/Brisbane) in 2011‐2012 and B/Yamagata virus for the next 4 seasons (B/Wisconsin in 2012‐2013, B/Massachusetts in 2013‐2014 through 2014‐2015, and B/Phuket in 2015‐2016).^17^, ^18^, ^19^, ^20^, ^21^ The quadrivalent vaccine was introduced in the 2013‐2014 season and added a B/Victoria (B/Brisbane) virus each subsequent year.

Each season, vaccination status was defined as receipt of at least one dose of any seasonal influenza vaccine (including for children < 9 years old), according to EMR, immunization registries, and/or plausible self/parental‐report (with estimated date and plausible location of vaccination). All sites used the same questionnaire to query enrollees about vaccination at enrollment. Vaccination status was confirmed using data requests from the EMR and state immunization registries (electronic records), followed by requests to health insurance plans and providers such as physicians' offices, work and community sites, and pharmacies. For the remaining unconfirmed vaccinations, plausible self‐report was based on additional information provided by the enrollee that had to include a location and approximate date.

### 2.3 Laboratory methods

Nasal and throat swabs were collected from participants ≥2 years old, and nasal swabs only were collected from children <2 years old. These specimens were tested for influenza using RT‐PCR with CDC‐provided primers and probes and were tested for A/subtype and B/lineage. Patients who tested positive for influenza were cases, and patients who tested negative for influenza were controls.

### 2.4 Statistical analysis

The influenza circulation period was calculated for each site in the Network. It was defined as the dates between the first and last influenza‐positive enrollment during each season. Participants enrolled outside the influenza circulation periods were excluded from analyses. The enrollment period details for the Network sites are shown in Table S2.

Baseline characteristics were compared across sites using chi‐square tests for categorical variables, and *t* tests or Wilcoxon rank‐sum statistics for continuous variables.

A test‐negative design was used to estimate VE by comparing the odds of vaccination among RT‐PCR confirmed influenza cases to the odds of vaccination among controls. Using odds ratios obtained from multivariable logistic regression models, VE estimates were calculated as VE = 100% × (1 − OR). A series of logistic regression models was conducted with RT‐PCR confirmed influenza A and influenza B, and the predominant circulating virus for each season as the dependent variable and vaccination status as the independent variable. The primary analyses determined VE for all influenza; subgroup analyses determined VE for influenza A\H1N1, influenza A\H3N2, influenza B Yamagata, and influenza B Victoria. The logistic regression models were adjusted a priori for age group (6 months‐4 years, 5‐17 years, 18‐49 years, 50‐64 years, ≥65 years), sex, race/ethnicity (non‐Hispanic white, non‐Hispanic black, non‐Hispanic other race, Hispanic any race), time from illness onset to enrollment (0‐2, 3‐4, 5‐7 days), presence of any high‐risk condition as determined by ICD‐9/10 codes, and calendar time (illness onset date in bi‐weekly intervals). Inclusion of these adjustment variables is comparable to those used by the US Flu VE Network for its VE estimations.^13^

Site‐specific differences in VE were estimated by four separate multivariable logistic regression models. For model 1, each site VE was independently estimated adjusting for age, sex, race/ethnicity, any high‐risk condition, interval from onset to enrollment, and calendar time. For model 2, overall VE combining all sites was estimated adjusting for the same variables used in model 1. For model 3, overall VE combining all sites was estimated as above, with a site variable added to adjust for possible confounding by study site. The site reference group was the Michigan site. Model 4 added site and vaccination status interaction to the variables in model 3. The vaccination status (two levels) and the clinical site (four levels) were represented in model 4 as categorical variables. The vaccination status by site interaction had four levels with three degrees of freedom and was declared as a classification variable in the model, with one being the reference category.

In addition to individual season estimates, data across seasons in which the vaccine viruses were identical to each other and matched the predominant viruses that circulated in the community, and for which there were sufficient numbers of cases to permit analysis, were combined for regression modeling with the site interaction term using model 4. Those seasons were 2013‐14 and 2015‐16 for A/H1N1 and 2013‐14 and 2014‐2015 for B/Yamagata. In seasons or combined seasons with statistically significant interactions, model 4 was used to examine the significance of vaccination status by site interaction. When there was a significant interaction indicated, then the vaccine effect of each site was compared with the vaccine effect of the reference site (Michigan). The probability values for vaccination status, clinical site, and the interaction were derived using Wald‐chi‐square test.

Further, the statistical heterogeneity of estimates across sites was assessed using *I* ^2^ statistic calculated from a random effects meta‐analysis.^22^ Estimates of *I^2^* > 50% were considered to be highly heterogeneous. Data were analyzed using SAS version 9.4 (SAS Institute). Statistical significance was set at *P* < .05.

## 3 RESULTS

**FIGURE 1:**
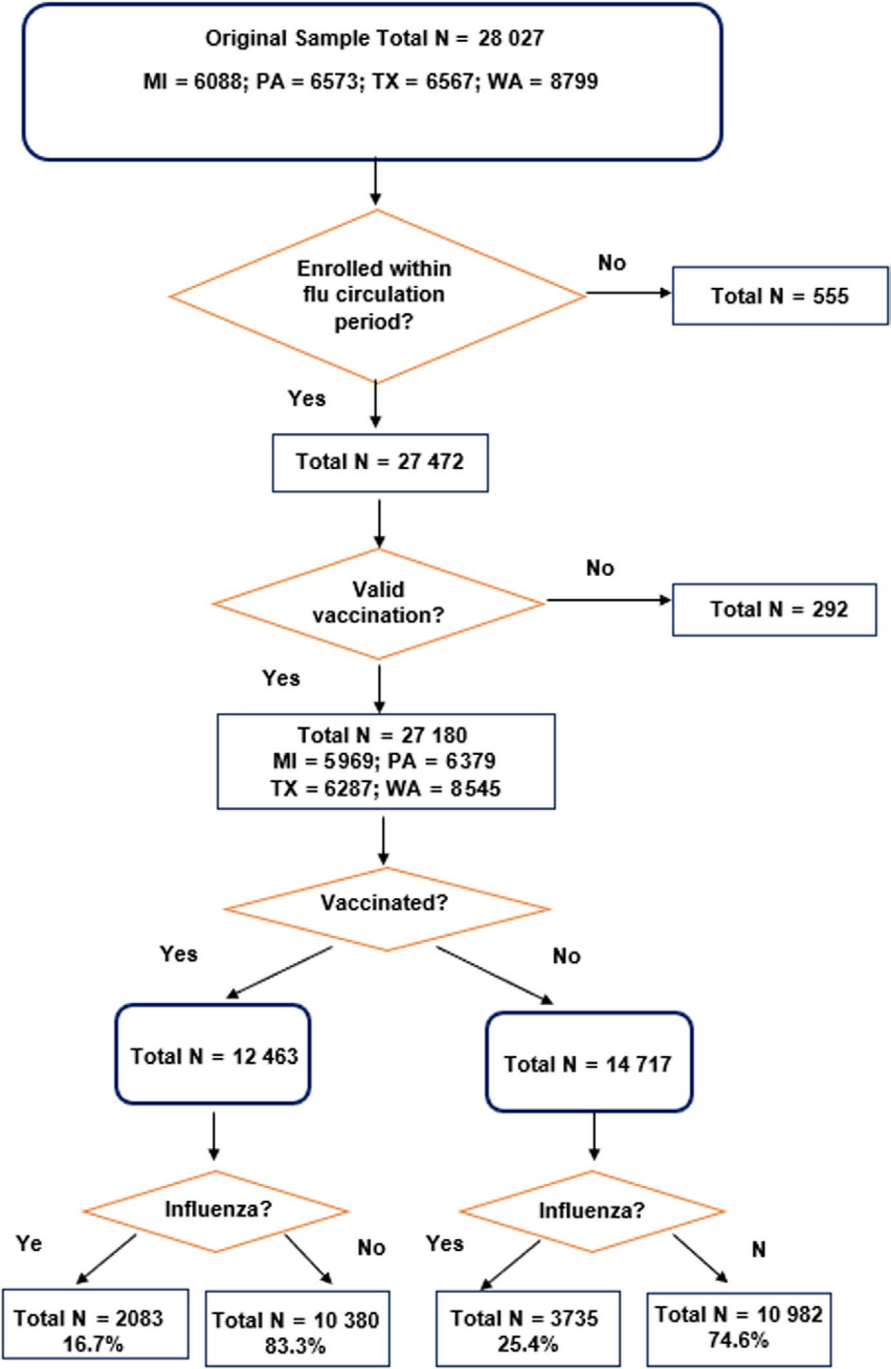
Enrollment Flow chart

There were 27 180 outpatients seeking care for ARI who were enrolled at the 4 US Flu VE Network sites within the annual circulation periods from January 12, 2012, to April 14, 2016, (Figure 1) after excluding 555 influenza‐negative patients (2%) who were enrolled outside those periods and the 292 (1%) who were vaccinated ≤14 days before illness onset. Twenty‐one percent (n = 2083) were influenza‐positive, and 79% (n = 21 362) were influenza‐negative. Of the influenza cases, 48% (n = 2770) were infected with A/H3N2, 26% (n = 1493) with A/H1N1 pdm09, 16.7% (n = 971) with B/Yamagata, and 7.3% (n = 426) with B/Victoria viruses. In addition, 92 influenza A viruses could not be subtyped, 41 influenza B viruses had no lineage identified due to high CT values, seven patients had influenza A/B co‐infections, and 18 patients' influenza results could not be confirmed. All individuals were included in the overall analyses, but those who were not subtyped were excluded from the subtype analyses.

Table S1 shows the circulating and vaccine viruses for each season. The predominant circulating strain varied from season to season but was identical across all Network sites for each season.

### 3.1 Enrollee characteristics

There were significant differences among sites (*P* < .001) across each demographic variable measured (Table 1). For example, the Washington site enrolled the lowest proportion of children (26%) and the highest proportion of senior adults (≥65 years; 17%), whereas the Michigan site enrolled the highest proportion of children (48%) and the lowest proportion of senior adults (8%), and the Pennsylvania site enrolled the largest proportion of working age adults (59%). Commensurate with this age distribution, the Washington site enrolled the highest proportion of participants with presence of a high‐risk condition (38%) and the highest vaccination rate (54%). More females than males enrolled in the study, and the racial/ethnic distribution of enrollees differed across sites, reflecting the racial/ethnic distributions of their respective geographical regions (*P* < .001). Michigan enrolled the highest proportion of non‐Hispanic black participants (21%), Washington enrolled the highest proportion of non‐Hispanics from other racial groups (16%), and Texas enrolled the highest proportion of Hispanic participants (24%). In the Pennsylvania and Michigan sites, approximately one‐quarter of enrollees were influenza cases compared with less than one‐fifth in Texas and Washington. Over 40% of participants were enrolled within 2 days of illness onset in Texas, between 3‐4 days in Michigan and Pittsburgh and 5‐7 days in Washington. Table S3 shows the various types of influenza vaccines used by the sites over the 5 years of the study that likely reflected availability in the market and purchasing decisions by the sites.

**TABLE 1:** Demographic characteristics, symptoms, and influenza status of enrollees in four US Flu VE Network sites over five influenza seasons, 2011‐2016

Characteristic	Michigan (N = 5969)	Pennsylvania (N = 6379)	Texas (N = 6287)	Washington (N = 8545)	*P* value
	N (%)	N (%)	N (%)	N (%)	
Age group					
6 mo‐4 y	1209 (20.2)	916 (14.4)	1008 (16.0)	978 (11.5)	<.001
5‐17 y	1634 (27.4)	1149 (18.0)	1726 (27.4)	1269 (14.9)	
18‐49 y	1663 (27.9)	2626 (41.2)	2103 (33.5)	3191 (37.3)	
50‐64 y	969 (16.2)	1141 (17.9)	873 (13.9)	1686 (19.7)	
≥65 y	494 (8.3)	547 (8.6)	577 (9.2)	1421 (16.6)	
Sex					
Male	2697 (45.2)	2630 (41.2)	2544 (40.5)	3788 (44.3)	<.001
Female	3272 (54.8)	3749 (58.8)	3743 (59.5)	4757 (55.7)	
Race/Ethnicity					
White, non‐Hispanic	3580 (60.2)	4973 (78.3)	4080 (64.9)	6163 (72.3)	<.001
Black, non‐Hispanic	1223 (20.6)	875 (13.8)	392 (6.2)	321 (3.8)	
Other race, non‐Hispanic	823 (13.8)	368 (5.8)	290 (4.6)	1379 (16.2)	
Hispanic, any race	321 (5.4)	132 (2.1)	1520 (24.2)	656 (7.7)	
Self‐rated general health status					
Excellent/Very good	4345 (73.0)	4367 (68.6)	4461 (71.0)	5874 (68.8)	<.001
Good	1246 (20.9)	1614 (25.3)	1402 (22.3)	2051 (24.0)	
Fair/Poor	362 (6.1)	389 (6.1)	421 (6.7)	612 (7.2)	
Any high‐risk condition	1966 (32.9)	2037 (31.9)	2133 (33.9)	3255 (38.1)	<.001
Presenting symptoms					
Fever	3373 (65.6)	3712 (58.2)	3353 (64.2)	4432 (63.8)	<.001
Fatigue	2100 (82.2)	4709 (73.8)	1737 (81.3)	2165 (91.6)	<.001
Sore Throat	3475 (67.6)	4265 (66.9)	3929 (76.5)	5174 (74.5)	<.001
Influenza status					
RT‐PCR negative	4497 (75.3)	4800 (75.2)	5038 (80.1)	7027 (82.2)	<.001
RT‐PCR positive	1472 (24.7)	1579 (25.8)	1249 (19.9)	1518 (17.8)	
Interval from onset to enrollment					
0‐2 d	2019 (33.8)	2140 (33.6)	2547 (40.7)	1814 (21.2)	<.001
3‐4 d	2398 (40.2)	2764 (43.4)	2312 (37.0)	3280 (38.4)	
5‐7 d	1552 (26.0)	1463 (23.0)	1395 (22.3)	3451 (40.4)	
Vaccinated	2655 (44.5)	2766 (43.4)	2436 (38.8)	4606 (53.9)	<.001

Table 2 shows influenza vaccination rates among cases and controls for the five seasons combined for each site. In all cases, overall or by vaccine subtype, the proportion of vaccinated controls exceeded the proportion of vaccinated cases, indicating some protection by the vaccine. Table 3 shows the number and percentage of cases caused by each virus strain by site, each season. There are noticeable differences among the sites most seasons. For example, in 2013‐14 in Michigan and Pennsylvania, 89%‐93% of cases were A/H1N1, whereas in Texas and Washington, 77%‐80% of cases were A/H1N1 with more of the remainder being B/Yamagata in these sites than in the former sites. In 2015‐2016, the differences were more marked with Michigan and Pennsylvania A/H1N1 cases comprising 69%‐70% of the total compared with 27%‐30% in Texas and Washington.

**TABLE 2:** Influenza vaccination of influenza controls, cases, and cases by strain/lineage in 4 US Flu VE Network sites, overall, and by subtype for 2011‐2016 combined

Site	Controls (% vaccinated)	Total cases (% vaccinated)	A/H1N1 cases (% vaccinated)	A/H3N2 cases (% vaccinated)	B/Yamagata cases (% vaccinated)	B/Victoria cases (% vaccinated)
Michigan	2128 (47)	537 (36)	115 (36)	290 (38)	80 (27)	26 (46)
Pennsylvania	2208 (46)	558 (35)	156 (28)	305 (40)	56 (37)	24 (35)
Texas	2080 (41)	356 (28)	62 (25)	180 (34)	69 (26)	35 (21)
Washington	3964 (56)	642 (42)	115 (32)	370 (52)	89 (35)	44 (33)

**TABLE 3:** Virus subtypes by season and site

Season/virus	Michigan N (%)	Pennsylvania N (%)	Texas N (%)	Washington N (%)	All sites
2011‐2012					
A/H1N1	4 (2.0)	5 (9.4)	41 (83.7)	55 (24.6)	105
A/H3N2	182 (90.1)	45 (84.9)	5 (10.0)	72 (32.1)	304
B Victoria	1 (0.5)	0 (0)	0 (0)	61 (27.2)	62
B Yamagata	13 (6.4)	2 (3.8)	2 (4.1)	34 (15.2)	51
A/B	2 (1.0)	1 (1.9)	1 (2.0)	2 (0.9)	6
Total	202	53	49	224	528
2012‐2013					
A/H1N1	14 (2.6)	8 (2.1)	12 (2.7)	16 (7.1)	50
A/H3N2	261 (48.2)	270 (71.4)	227 (51.0)	178 (79.5)	936
B Victoria	20 (3.7)	28 (7.4)	44 (9.9)	6 (2.7)	98
B Yamagata	238 (44.0)	49 (13.0)	158 (35.5)	5 (2.2)	450
A/B	8 (1.5)	23 (6.1)	4 (0.9)	19 (8.5)	54
Total	541	378	445	224	1588
2013‐2014					
A/H1N1	129 (89.6)	288 (92.9)	144 (77.4)	196 (79.7)	757
A/H3N2	8 (5.5)	8 (2.6)	7 (3.8)	7 (2.9)	30
B Victoria	1 (0.7)	1 (0.3)	10 (5.4)	3 (1.2)	15
B Yamagata	4 (2.8)	8 (2.6)	17 (9.1)	23 (9.3)	52
A/B	2 (1.4)	5 (1.6)	8 (4.3)	17 (6.9)	32
Total	144	310	186	246	886
2014‐2015					
A/H1N1	1 (0.3)	0 (0)	0 (0)	3 (0.6)	4
A/H3N2	318 (92.7)	434 (90.6)	284 (74.5)	424 (82.3)	1460
B Victoria	1 (0.3)	7 (1.5)	25 (6.6)	13 (2.5)	46
B Yamagata	17 (5.0)	33 (6.9)	61 (16.0)	68 (13.2)	179
A/B	6 (1.7)	5 (1.0)	11 (2.9)	7 (1.4)	29
Total	343	479	381	515	1718
2015‐2016					
A/H1N1	177 (69.1)	258 (69.7)	53 (26.9)	98 (30.0)	586
A/H3N2	11 (4.3)	9 (2.4)	18 (9.1)	38 (11.6)	76
B Victoria	33 (12.9)	32 (8.6)	91 (46.2)	50 (15.3)	206
B Yamagata	22 (8.6)	60 (16.2)	31 (15.7)	128 (39.1)	241
A/B	13 (4.9)	11 (3.0)	4 (2.0)	13 (4.0)	41
Total	256	370	197	327	1150

Table 4 shows VE estimates for all enrollees combined for all influenza types and by the predominant circulating strains for each season. For all sites combined, VE was statistically significant every season against all influenza and against the predominant circulating strains (Model 2). Statistically significant VE was observed in at least one site during each of the study years. Each site had at least one year in which the vaccine was ineffective against the predominant strain.

**TABLE 4:** Adjusted vaccine effectiveness against medically attended influenza in 4 US Flu VE Network sites by season (2011‐2016) and predominant virus subtype

Season	MODEL 1^a^				MODEL 2^a^	MODEL 3^b^	MODEL 4^c^ *P* value for interaction^e^
	Adjusted^d^ VE, % (95% CI)				Overall	Overall with site added	
	Michigan	Pennsylvania	Texas	Washington			
*2011‐2012*							
Overall	**46 (23, 62)**	38 (−14, 66)	**71 (37, 86)**	**55 (37, 68)**	**52 (40, 61)**	**53 (41, 62)**	.434
A/H3N2	**42 (13, 60)**	32 (−29, 65)	66 (−333, 97)^f^	**44 (5, 62)**	**42 (24, 55)**	**40 (21, 54)**	.854
B/Victoria	—^g^	—^g^	—^g^	**62 (29, 80)**	**56 (20, 75)**	—^g^	—^g^
B/Yamagata	87 (−4, 98)	—^g^	—^g^	49 (−7, 76)	**61 (26, 80)**	**65 (33, 82)**	—^g^
*2012‐2013*							
Overall	**52 (39, 62)**	**36 (17, 51)**	**56 (43, 66)**	**51 (31, 65)**	**50 (43, 56)**	**49 (42, 55)**	.136
A/H3N2	**46 (27, 61)**	**36 (13, 53)**	**50 (31, 64)**	**37 (9, 57)**	**42 (32, 50)**	**43 (33, 51)**	.491
B/Yamagata	**65 (50, 75)**	13 (−71, 55)	**58 (38, 72)**	—^g^	**63 (54, 71)**	**59 (48, 67)**	—
*2013‐2014*							
Overall	**58 (34, 73)**	**38 (14, 55)**	**45 (19, 63)**	**59 (44, 70)**	**51 (42, 59)**	**51 (41, 59)**	.215
A/H1N1	**63 (40, 78)**	**41 (18, 58)**	32 (−8, 57)	**63 (48, 74)**	**53 (43, 61)**	**52 (42, 61)**	.088
B/Yamagata	—^g^	—^g^	**75 (8, 93)**	34 (−56, 72)	**47 (3, 71)**	**51 (9, 74)**	.911
*2014‐2015*							
Overall	15 (−10, 35)	**36 (17, 50)**	5 (−23, 26)	**19 (0, 35)**	**19 (9, 28)**	**20 (10, 29)**	.124
A/H3N2	17 (−9, 37)	**33 (17, 49)**	‐6 (−44, 23)	11 (−13, 29)	**14 (2, 25)**	**16 (5, 27)**	.115
B/Yamagata	−18 (−232, 58)	48 (−11, 76)	29 (−26, 60)	**58 (28, 76)**	**42 (19, 58)**	**39 (16, 56)**	.298
*2015‐2016* ^h^							
Overall	24 (−4, 44)	**40 (22, 54)**	**53 (33, 67)**	**61 (49, 71)**	**44 (35, 51)**		**.007**
Vaccination status × Site (ref., = Michigan)					Pennsylvania	14 (−26, 42)	.438
					Texas	**39 (4, 61)**	**.034**
					Washington	**47 (20, 64)**	**.002**
A/H1N1	18 (−17, 43)	**42 (21, 57)**	**53 (12, 75)**	**64 (43, 78)**	**39 (26, 50)**	**43 (31, 53)**	.091
B/Yamagata	39 (−51, 75)	43 (−2, 69)	**57 (0, 81)**	**66 (49, 78)**	**56 (41, 67)**	**57 (43, 68)**	.321
*2011‐2016*							
Overall	**38 (29, 45)**	**36 (27, 43)**	**42 (33, 49)**	**47 (41, 53)**	**40 (37, 44)**	**41 (37, 44)**	.205
*2013‐2014 and 2015‐2016 (same predominant A strain)*							
A/H1N1	**38 (19, 53)**	**44 (30, 55)**	**40 (14, 58)**	**62 (51, 71)**	**48 (40, 54)**		**.025**
Vaccination status × Site (ref., = Michigan)					Pennsylvania	10 (−25, 36)	.531
					Texas	19 (−24, 47)	.338
					Washington	**41 (15, 59)**	**.005**
*2013‐2014 and 2014‐2015 (same predominant B lineage)*							
B/Yamagata	−7 (−168, 57)	41 (−16, 70)	**40 (0, 64)**	**52 (25, 70)**	**42 (23, 52)**	**41 (21, 56)**	.511

Note

Bold indicates significant vaccine effectiveness and significant interaction effect.

Regression models:

^a^ Base case: logit(*y*) = *β*_0_ + *β*_1_*vaccination status + *β*_2_*age + *β*_3_*sex + *β*_4_*race/ethnicity + *β*_5_*any high‐risk condition + *β*_6_*interval from onset to enrollment + *β*_7_*calendar time + error.

^b^ With site added: logit(*y*) = *β*_0_ + *β*_1_*vaccination status + *β*_2i_*Clinical site + *β*_3_*age + *β*_4_*sex + *β*_5_*race/ethnicity + *β*_6_*any high‐risk condition + *β*_7_*interval from onset to enrollment + *β*_8_*calendar time + error; *i* = site 1,2,3,4.

^c^ Interaction: logit(*y*) = *β*_0_ + *β*_1_*vaccination status + *β*_2i_*Clinical site + *β*_3j_*(vaccination status × Clinical site) + *β*_4_*age + *β*_5_*sex + *β*_6_*race/ethnicity + *β*_7_*any high‐risk condition + *β*_8_*interval from onset to enrollment + *β*_9_*calendar time + error; *i* = site 1,2,3,4; *j* = 1,2,3,4 site*vaccination status.

^d^ Multivariable logistic regression models adjusted for age, sex, race/ethnicity, any high‐risk condition, interval from onset to enrollment, and calendar time(bi‐week). Race/ethnicity: Combined black, non‐Hispanic, other race, non‐Hispanic, and Hispanic any race into non‐white.

^e^ *P*‐value for interaction: (site × vaccination status).

^f^ Sex is excluded from the adjusted model due to <5 in cell frequencies, and the validity of the model fit was questionable when included.

^g^ Insufficient data for analysis or convergence of the model.

^h^ Similar data were originally published in Ref. [16] (Table S2), which included a 5th site, Wisconsin.

When site was added to the model (Model 3), VE estimates were similar to the model without site included (Table 4, column 7), indicating that site was not a confounder. To test for effect modification in the site differences, an interaction term was added to the model. Among the five seasons tested, the interaction term was significant only in 2015‐2016 (*P* for the interaction term = .007). Individual comparisons of VE estimates between the sites revealed that the Washington and Texas sites had significantly higher overall VE (61%; *P* = .002 and 53%; *P* = .034, respectively) than the reference site, that is, the site with the lowest VE for that season, Michigan (VE = 24%).

**FIGURE 2:**
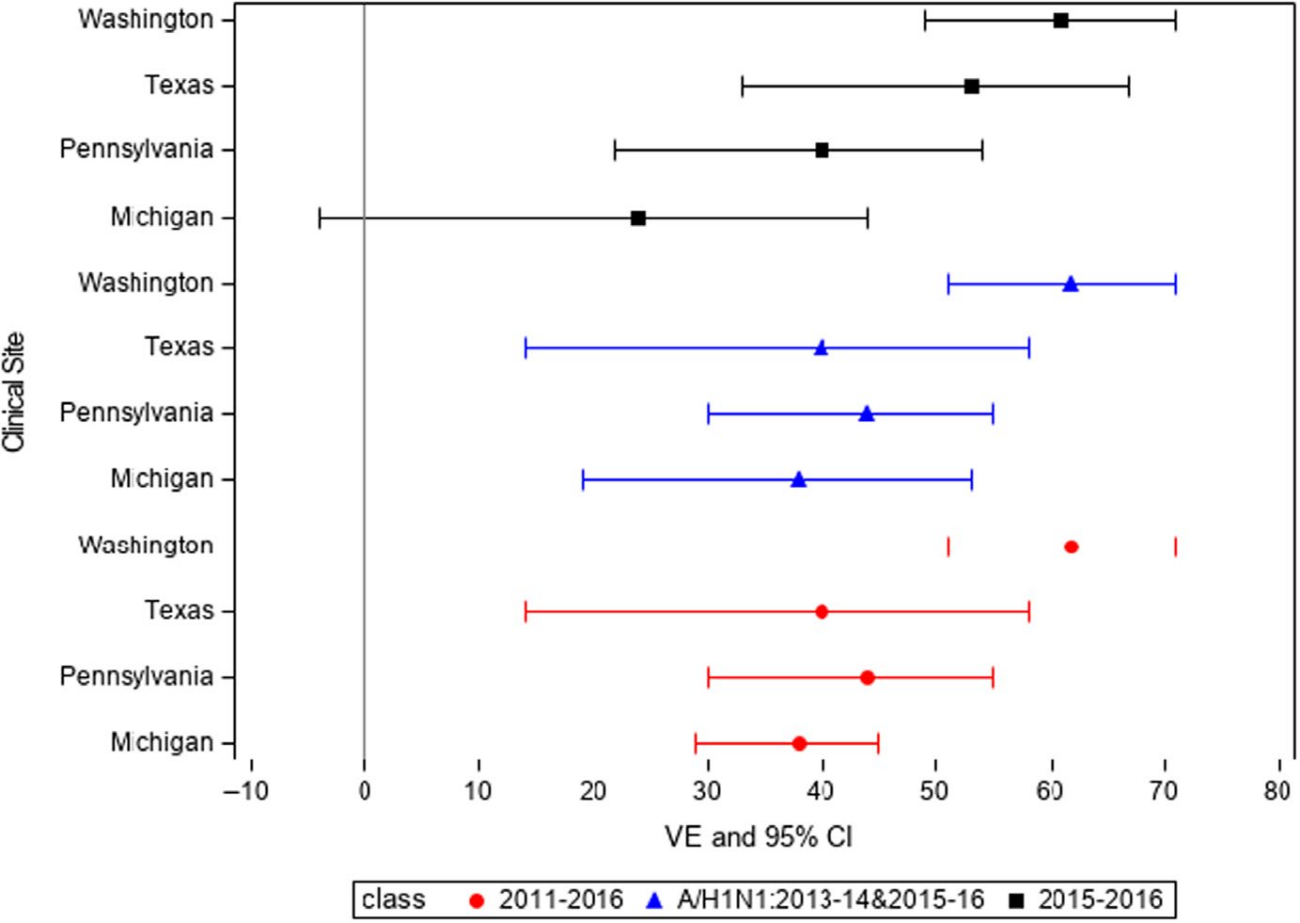
Vaccine effectiveness by site

Two analyses combined data from seasons in which the vaccine viruses were identical, the predominant A and B viruses that circulated in the community were identical, and there were sufficient numbers of cases to permit analysis. Those seasons were 2013‐14 and 2015‐16 for A/H1N1 and 2013‐14 and 2014‐2015 for B/Yamagata. For A/H1N1, VE was 42% (95% CI = 28, 53; *P* for interaction = .025). Although all sites had significant VE against A/H1N1, individual comparisons of VE estimates between the sites revealed that the Washington site had significantly higher VE (65%) than the site with the lowest VE, Michigan (38%; *P* = .013). For B/Yamagata, VE was 42% (95% CI = −8, 72; *P* for interaction = 0.511). VE point estimates and confidence intervals for all seasons combined, for A/H1N1 for 2013 = 2014 and 2015‐2016, and for all influenza for 2015‐2016, are shown in Figure 2.

In meta‐analysis testing for heterogeneity across the four clinical sites, three comparisons were made. Results are shown in Table S4. Consistent with the results reported in Table 4 for 2011‐2016 overall VE, no heterogeneity of VE was observed (chi‐square = 5.95, *P* = .114, *I* ^2^ = 49.6%). By contrast, the subgroup analysis of two seasons (2013‐14 and 2015‐16) with predominant A/H1N1 strain and overall analysis for one season 2015‐2016 revealed that there is significant variation present among the clinical sites in VE with high heterogeneity (chi‐square = 8.0, *P* = .046, *I* ^2^ = 62.5% for combined 2013‐2014 and 2015‐2016 A/H1N1 and chi‐square = 11.91, *P* = .008, *I* ^2^ = 74.8% for 2015‐2015 overall VE). This heterogeneity was thought to have occurred because of the Washington site's differences in demographic characteristics and vaccination status. A sensitivity analysis was performed excluding the Washington site. The results revealed no heterogeneity for the combined 2013‐2014 and 2015‐2016 seasons' A/H1N1 analysis (chi‐square = 0.3, *P* = .861, *I* ^2^ = 0%). For the 2015‐2016 season overall VE, the heterogeneity declined to *I* ^2^ = 51.7%, suggesting that the Washington site might be driving the observed differences.

## 4 DISCUSSION

During the 2011‐2016 influenza seasons, the influenza vaccine provided moderate protection against influenza illness for which the patient sought outpatient care, as reported by the CDC's Influenza VE Network: 47% in 2011‐2012; 49% in 2012‐2013; 54% in 2013‐2014; 19% in 2014‐2015; and 48% in 2015‐2016.^11^, ^12^, ^13^, ^14^, ^16^ In two seasons, influenza A/H1N1 predominated, and in three seasons, A/H3N2 predominated. The vaccine was effective across all four of the Network sites in two seasons (2012‐2013 and 2013‐2014). In the other three seasons, the vaccine was effective against all influenza in three sites in 2011‐2012 and in 2015‐2016, and two of the sites in 2014‐2015. When site was included as an interaction term in regression models, VE against all influenza varied by site in only one season (2015‐2016) and VE against A/H1N1 varied by site when the 2013‐2014 and 2015‐2016 seasons were combined. These differences are not likely due to methodological differences, as a standard protocol was used across all sites, but may be attributed to differences in these geographically dispersed sites.

Geographical differences in the epidemiology of seasonal influenza have been reported. For example, in Latin America, the timing of the annual influenza epidemic varies with geographical location, that is, in the temperate Southern Hemisphere, the primary circulation period is May through October, whereas influenza circulates all year in tropical areas.^23^ In large countries like Brazil^23^ or the USA,^24^ influenza peaks at different times in different regions. However, studies from Japan^25^ and Saudi Arabia^26^ have reported regional differences in incidence of influenza. In small countries especially those with similar geography like Norway, Sweden, and Denmark, similar seasonal epidemics have been reported. Spread of influenza was attributed to population size within these smaller countries when they were geographically similar, but not between those with differing geography.^24^ For example, in the 2018‐2019 season, Europe reported co‐circulation of influenza A/H3N2 and A/H1N1^27^ with varying proportions of these strains reported across countries. Comparing the spread of influenza across geographical regions has been recommended to increase understanding of the complex mechanisms of disease dynamics,^24^ and in turn, may contribute to better approaches to influenza prevention and mitigation.

Genetic sequencing of influenza viruses has the potential to increase our understanding of the causes of differential VE. In 2014‐15, most of the A/H3N2 viruses circulating in the USA were antigenically different from the A/H3N2 vaccine virus for that season, except in the Pennsylvania site where a mixture of A/H3N2 clades occurred; correspondingly, only that site reported significant VE against A/H3N2 in 2014‐15.^15^ Geographical differences among the sites might reflect the distribution of various mutations and contribute to potential differences in vaccine effectiveness.

It is possible that differences in VE across sites are related to the types of vaccine used at any given site. During the time of this study, there were fewer options for vaccine types than are currently widely available; for example, cell‐based vaccines, first available in 2016, represented only 10%‐15% of the US market in 2017‐2018^28^ and recombinant vaccines were licensed in 2017 after this study. In addition, during this time period, LAIV was recommended, preferred, and not recommended.^29^ In the sites participating in this study, the use of trivalent standard‐dose vaccine was declining, while quadrivalent standard‐dose vaccine was increasing, and high dose vaccine use was increasing despite its higher cost. The Washington site did not report any high dose vaccine among enrollees until 2015‐2016, whereas other sites began to report its use as early as 2013‐2014.

Enrollees in this study of sites located hundreds to thousands of miles apart represent unique combinations of population densities, demographic characteristics, topography, modes of transportation, types of vaccines received, and perhaps vaccine coverage. Any or all of these factors might contribute to influenza epidemiology and vaccine effectiveness. However, larger sample sizes may be necessary to elucidate those relationships. Further studies of differences in vaccine effectiveness by region may provide better estimates for computational modeling studies^10^ that are used to predict influenza epidemiology, burden, and anticipated resource needs.

### 4.1 Strengths and limitations

Strengths of this study include using data from five influenza seasons with different circulating strains, inclusion of diverse geographical sites, and generally adequate sample size. The test‐negative design is subject to potential biases such as misclassification of the exposure variable (vaccination status), confounding or effect modification by prior vaccination effects, and confounding by characteristics of study enrollees; indeed, our sites differed in demographic characteristics and vaccination rates. However, consistent protocols and rigorous attempts by the sites to confirm vaccination status were employed to minimize bias. Insufficient numbers of circulating viruses in some sites in some seasons limited some VE estimates. Had all five US Flu VE network sites participated, we may not have had low numbers for some analyses. It is possible that the differences observed may be attributed to unaccounted for differences among the Network sites. Vaccine type differed among sites and may affect overall VE. In addition, the effects of prior infection or prior seasons' vaccination are unknown.

## 5 CONCLUSIONS

With few exceptions, site‐specific vaccine effectiveness estimates aligned with each other and overall VE estimates. Observed VE differences may reflect inherent differences in community characteristics of the participating sites and highlight the importance of diverse settings for studying influenza vaccine effectiveness.

## CONFLICTS OF INTEREST

RKZ has/had a research grant from Sanofi Pasteur, Inc RKZ and MPN have/had research grant funding from Merck & Co, Inc and from Pfizer, Inc MJG has received institutional research grant from AstraZeneca‐MedImmune. MLJ reports research funding from Sanofi Pasteur, unrelated to the present work. ASM reports consulting fees from Sanofi, Seqirus, and Roche. ETM reports consulting fees from Pfizer. The other authors have no conflicts to report.

## AUTHOR CONTRIBUTIONS

**Goundappa K. Balasubramani**: Conceptualization‐Equal, Formal analysis‐Equal, Writing‐review & editing‐Lead. **Mary Patricia Nowalk**: Project administration‐Equal, Writing‐original draft‐Equal, Writing‐review & editing‐Equal. **Theresa Sax**: Data curation‐Equal, Writing‐review & editing‐Equal. **Joe Suyama**: Project administration‐Equal, Writing‐review & editing‐Equal. **Emily Bobyock**: Data curation‐Equal, Formal analysis‐Equal, Writing‐review & editing‐Equal. **Charles R. Rinaldo**: Conceptualization‐Equal, Funding acquisition‐Equal, Investigation‐Equal, Methodology‐Equal, Project administration‐Equal. **Emily Martin**: Data curation‐Equal, Project administration‐Equal, Writing‐review & editing‐Equal. **Arnold Monto**: Conceptualization‐Equal, Funding acquisition‐Equal, Investigation‐Equal, Writing‐review & editing‐Equal. **Michael Jackson**: Conceptualization‐Equal, Data curation‐Equal, Funding acquisition‐Equal, Methodology‐Equal, Project administration‐Equal, Writing‐review & editing‐Equal. **Manjusha Gaglani**: Conceptualization‐Equal, Funding acquisition‐Equal, Investigation‐Equal, Methodology‐Equal, Project administration‐Equal, Writing‐review & editing‐Equal. **Brendan Flannery**: Conceptualization‐Equal, Investigation‐Equal, Methodology‐Equal, Project administration‐Equal, Writing‐review & editing‐Equal. **Jessie Chung**: Data curation‐Equal, Formal analysis‐Equal, Writing‐review & editing‐Equal. **Richard Zimmerman**: Conceptualization‐Equal, Funding acquisition‐Equal, Investigation‐Equal, Project administration‐Equal, Writing‐review & editing‐Equal.

## Supporting information

Supinfo
