# Supplementary material for: Influenza vaccine effectiveness among outpatients in the US Influenza Vaccine Effectiveness Network by study site 2011‐2016
Source: Influenza Other Respir Viruses. 2020 Apr 16;14(4):380–90. doi: 10.1111/irv.12741 (PMC7298285; doi:10.1111/irv.12741)
Supplement: Supplementary file 1 — Supinfo [file IRV-14-380-s001.docx]

| **Supplemental Table 1. Influenza viruses contained in the influenza vaccine and that circulated* in the Northern Hemisphere for 2011-2016 by season.** | | | | | | |
| --- | --- | --- | --- | --- | --- | --- |
| **Strain/ lineage** | **Vaccine or Circulating** | **2011-2012^18^** | **2012-2013^19^** | **2013-2014^20^** | **2014-2015^21^** | **2015-2016^22^** |
| A/H1N1 | Both | A/California/7/2009 | A/California/7/2009 | **A/California /7/2009** | A/California/7/2009 | **A/California/7/2009** |
| A/H3N2 | Vaccine | **A/Perth/16/2009** | **A/Victoria/361/2011** | A/Texas/50/2012 | **A/Texas/50/2012** | A/Switzerland/ 9715293/2013 |
| A/H3N2 | Circulating | A/Perth/16/2009 | A/Victoria/361/2011 | A/Texas/50/2012 | A/Texas/50/2012 and  A/Switzerland/ 9715293/2013 | A/Switzerland/ 9715293/2013 |
| B Victoria | Both | B/Brisbane/60/2008 | B/Brisbane/60/2008 | B/Brisbane/60/2008 | B/Brisbane/60/2008 | B/Brisbane/60/2008 |
| B Yamagata | Both | -- | B/Wisconsin/1/2010^‡^ | B/Massachusetts/ 2/2012^‡^ | B/Massachusetts/ 2/2012^‡^ | B/Phuket/3073/2013^‡^ |

*Circulated in sufficient numbers for analysis

^‡^B lineage contained in the trivalent vaccine

| **Supplemental Table 2. Influenza circulation period for four US VE Network sites, 2011-2016, by site and year** | | | | | | |
| --- | --- | --- | --- | --- | --- | --- |
| **Season** | **Enrollment period** | **Total enrolled, n** | **Influenza circulation period** | **Days of enrollment during influenza circulation, n** | **Enrolled during influenza circulation, n** | **Influenza PCR positive**  **during circulation period, n** |
|  |  |  |  |  |  |  |
| Michigan | | | | | | |
| 2011-2012 | 1/9/2012 – 4/5/2012 | 1,227 | 1/12/2012 – 4/5/2012 | 85 | 1,205 | 202 |
| 2012-2013 | 12/17/2012 – 3/29/2013 | 1,375 | 12/17/2012 – 3/29/2013 | 103 | 1,375 | 541 |
| 2013-2014 | 12/9/2013 – 3/7/2014 | 836 | 12/9/2013 – 3/4/2014 | 86 | 826 | 144 |
| 2014-2015 | 11/10/2014 – 3/5/2015 | 1,563 | 11/10/2014 – 3/4/2015 | 115 | 1,562 | 343 |
| 2015-2016 | 1/4/2016 – 4/14/2016 | 1,087 | 1/5/2016 – 4/14/2016 | 101 | 1,072 | 256 |
| Total |  | 6,088 |  | 490 | 6,040 | 1,486 |
| Pennsylvania | | | | | | |
| 2011-2012 | 1/17/2012-4/27/2012 | 740 | 1/24/2012-4/25/2012 | 93 | 694 | 53 |
| 2012-2013 | 12/3/2012-3/28/2013 | 1,171 | 12/3/2012-3/28/2013 | 116 | 1,171 | 378 |
| 2013-2014 | 12/2/2013-3/23/2014 | 1,207 | 12/4/2013-3/23/2014 | 110 | 1,198 | 310 |
| 2014-2015 | 11/24/2014-3/11/2015 | 1,584 | 11/28/2014-3/10/2015 | 103 | 1,569 | 479 |
| 2015-2016 | 11/18/2015-4/15/2016 | 1,871 | 12/1/2015-4/14/2016 | 136 | 1,813 | 370 |
| Total |  | 6,573 |  | 558 | 6,445 | 1,590 |
| Texas | | | | | | |
| 2011-2012 | 1/19/2012 – 4/5/2012 | 803 | 1/30/2012 – 4/4/2012 | 66 | 709 | 49 |
| 2012-2013 | 12/9/2012 – 3/20/2013 | 1,445 | 12/09/2012 – 3/28/2013 | 110 | 1,445 | 445 |
| 2013-2014 | 12/10/2013 – 4/16/2014 | 1,035 | 12/10/2013 – 4/16/2014 | 128 | 1,035 | 186 |
| 2014-2015 | 11/13/2014 – 4/10/2015 | 1,783 | 11/13/2014 –4/8/2015 | 147 | 1,762 | 381 |
| 2015-2016 | 11/2/2015 – 4/10/2016 | 1,501 | 12/17/2015 – 4/9/2016 | 115 | 1,389 | 197 |
| Total |  | 6,567 |  | 566 | 6,340 | 1,258 |
| Washington | | | | | | |
| 2011-2012 | 1/17/2012 – 4/14/2012 | 1,288 | 1/26/2012 – 4/14/2012 | 80 | 1,224 | 224 |
| 2012-2013 | 1/2/2013 – 4/6/2013 | 1,199 | 1/2/2013 – 4/2/2013 | 91 | 1,169 | 224 |
| 2013-2014 | 12/9/2013 – 4/11/2014 | 1,629 | 12/10/2013 – 4/11/2014 | 123 | 1,621 | 246 |
| 2014-2015 | 12/1/2014 – 4/3/2015 | 2,841 | 12/1/2014 – 4/3/2015 | 124 | 2,841 | 515 |
| 2015-2016 | 11/30/2015 – 4/15/2016 | 1,842 | 12/3/2015 – 4/15/2016 | 135 | 1,792 | 327 |
| Total |  | 8,799 |  | 553 | 8,647 | 1,536 |

| **Supplemental Table 3. Distribution of type of vaccines used for enrollees by season and clinical site** | | | | | |
| --- | --- | --- | --- | --- | --- |
| **Season** | **Type of Vaccine, n (%)** | | | | |
|  | **LAIV** | **Standard dose - Trivalent** | **Standard dose - Quadrivalent** | **High dose - Trivalent** | **Unknown type** |
| Michigan | | | | | |
| 2011-2012 | 43 (21.2) | 422 (36.8) | 0 (0.0) | 0 (0.0) | 0 (0.0) |
| 2012-2013 | 38 (18.7) | 519 (45.2) | 0 (0.0) | 6 (7.1) | 0 (0.0) |
| 2013-2014 | 16 (7.9) | 125 (10.9) | 176 (14.5) | 2 (2.3) | 7 (100.0) |
| 2014-2015 | 75 (36.9) | 53 (4.6) | 585 (48.3) | 44 (51.8) | 0 (0.0) |
| 2015-2016 | 31 (15.3) | 29 (2.5) | 451 (37.2) | 33 (38.8) | 0 (0.0) |
| Total | 203 | 1,148 | 1,212 | 85 | 7 |
| Pennsylvania | | | | | |
| 2011-2012 | 29 (13.5) | 292 (22.5) | 0 (0.0) | 0 (0.0) | 0 (0.0) |
| 2012-2013 | 39 (18.1) | 531 (40.9) | 0 (0.0) | 0 (0.0) | 0 (0.0) |
| 2013-2014 | 32 (14.9) | 247 (19.0) | 5 (0.6) | 0 (0.0) | 117 (34.8) |
| 2014-2015 | 76 (35.4) | 154 (11.9) | 337 (38.9) | 8 (15.4) | 127 (37.8) |
| 2015-2016 | 39 (18.1) | 73 (5.6) | 524 (60.5) | 44 (84.6) | 92 (27.4) |
| Total | 215 | 1,297 | 866 | 52 (1.9) | 336 |
| Texas | | | | | |
| 2011-2012 | 21 (9.0) | 275 (27.4) | 0 (0.0) | 0 (0.0) | 0 (0.0) |
| 2012-2013 | 91 (38.9) | 460 (45.8) | 0 (0.0) | 1 (2.3) | 0 (0.0) |
| 2013-2014 | 36 (15.4) | 158 (15.7) | 156 (14.0) | 6 (13.9) | 19 (45.2) |
| 2014-2015 | 63 (26.9) | 85 (8.5) | 471 (42.3) | 14 (32.6) | 16 (38.1) |
| 2015-2016 | 23 (9.8) | 26 (2.6) | 486 (43.7) | 22 (51.2) | 7 (16.7) |
| Total | 234 | 1,004 | 1,113 | 43 | 42 |
| Washington | | | | | |
| 2011-2012 | 38 (10.3) | 562 (20.3) | 0 (0.0) | 0 (0.0) | 0 (0.0) |
| 2012-2013 | 34 (9.2) | 608 (22.0) | 0 (0.0) | 0 (0.0) | 0 (0.0) |
| 2013-2014 | 45 (12.2) | 779 (28.2) | 1 (0.1) | 0 (0.0) | 58 (96.7) |
| 2014-2015 | 197 (53.4) | 701 (25.4) | 703 (54.0) | 0 (0.0) | 2 (3.3) |
| 2015-2016 | 55 (14.9) | 114 (4.1) | 598 (45.9) | 111 (100.0) | 0 (0.0) |
| Total | 369 | 2,764 | 1,302 | 111 | 60 |

| **Supplemental Table 4. Heterogeneity through Meta-Analysis** | | | |
| --- | --- | --- | --- |
| **Heterogeneity Tests** | **2015-2016**  **Overall** | **2011-2016**  **Overall** | **2013-2014 and 2015-2016 Predominant A/H1N1 strain** |
| Moment Estimate (τ^2^) | 2.97 | 1.49 | 1.99 |
| Test of Homogeneity (Q) | 11.91 | 5.95 | 7.99 |
| Degrees of freedom | 3 | 3 | 3 |
| P value | 0.008 | 0.114 | 0.046 |
| Overall heterogeneity (I^2^) | 74.8% | 49.6% | 62.5% |
| Test for overall effect | 3.45 | 2.44 | 2.83 |
| *P* value using Z test | <.001 | 0.007 | 0.002 |
